# Complete Genome Sequence of *Shewanella* sp. Strain Lzh-2, an Algicidal Bacterial Strain Isolated from Lake Taihu, People’s Republic of China

**DOI:** 10.1128/MRA.00339-21

**Published:** 2021-04-29

**Authors:** Zhenghua Li, Feng Song, Mei Chen

**Affiliations:** a Shandong Provincial Key Laboratory of Biophysics, Institute of Biophysics, Dezhou University, Dezhou, People’s Republic of China; b School of Bioengineering, Qilu University of Technology, Shandong Academy of Sciences, Jinan, People’s Republic of China; University of Delaware

## Abstract

*Shewanella* sp. strain Lzh-2 is an algicidal bacterium isolated from surface water samples collected from Meiliang Bay of Lake Taihu in China. Here, we present the complete genome sequence of *Shewanella* sp. Lzh-2. Some functional genes and secondary metabolite gene clusters were predicted.

## Abstract (precis)

*Shewanella* sp. strain Lzh-2 is an algicidal bacterium isolated from surface water samples collected from Meiliang Bay of Lake Taihu. Here, we present the complete genome sequence of *Shewanella* sp. Lzh-2. Some functional genes and secondary metabolite gene clusters were predicted.

## ANNOUNCEMENT

*Shewanella* sp. strain Lzh-2 was isolated from surface water samples collected from Meiliang Bay of Lake Taihu in China (1). Strain Lzh-2 exhibited significant algicidal activity toward several cyanobacterial species (1). Two algicidal substances (hexahydropyrrolo[1,2-*a*]pyrazine-1,4-dione and 2,3-indolinedione) have been purified from the culture of strain Lzh-2, and they both possessed strong algicidal activity against Microcystis aeruginosa, which was reported to be one of the most dominant microorganisms in the cyanobacterial blooms of Lake Taihu (1–3). Overall, the strain Lzh-2 and its two algicidal secretions have potential applications in controlling cyanobacterial blooms.

*Shewanella* sp. Lzh-2 was incubated in beef extract peptone liquid medium (beef extract, 3 g/liter; peptone, 10 g/liter; agar, 15 g/liter [pH 7.4 to 7.6]) and cultivated with a shaking speed of 200 rpm at 28°C for 48 h. Genomic DNA was extracted using the cetyltrimethylammonium bromide (CTAB) method (4). The DNA libraries were prepared using a SMRTbell template prep kit 1.0 (PacBio, USA), and the DNA fragments generated were tested with an Agilent Bioanalyzer 2100. The fragments longer than 10 kb were selected using BluePippin (Sage Science, USA) and sequenced using the PacBio Sequel platform.

The whole-genome sequencing generated 518,801 reads (*N*_50_, 9,414 bp), and the reads were then filtered and assembled into a single scaffold using the Hierarchical Genome Assembly Process (HGAP) (5) and Canu (6). The genome was circular and consisted of a 4,634,534-bp chromosome with a GC content of 46.31%. A total of 4,023 open reading frames (ORF) were predicted in the genome using GeneMarkS (7), accounting for 85.85% of the length of the whole genome. Analysis with SignalP 5.0 (http://www.cbs.dtu.dk/services/SignalP/) (8) showed that 388 of the 4,023 genes encoded secretory proteins. Genes encoding carbohydrate-active enzymes (CAZymes) were annotated using the hmmscan tool (9). There were 106 genes encoding predicted carbohydrate enzymes, which comprised 34 glycoside hydrolases, 23 glycosyl transferases, 5 polysaccharide lyases, 23 carbohydrate esterases, 11 auxiliary activities, and 10 carbohydrate-binding modules.

Five secondary metabolite gene clusters were predicted using antiSMASH 5.0 (https://antismash.secondarymetabolites.org/) (10). The five gene clusters belonged to five different types of secondary metabolite clusters, including an aryl polyene type cluster (chromosomes [chr.] 342896 to 386750) synthesizing aryl polyenes (11), a siderophore type cluster (chr. 1663845 to 1675737) synthesizing desferrioxamine E (12), a RiPP-like type cluster (chr. 2627252 to 2638091), a beta-lactone type cluster (chr. 2734421 to 2765790) synthesizing eicosapentaenoic acid (13), and a ghlE-KS type cluster (chr. 3025122 to 3081331) synthesizing plipastatin (14). The two algicidal compounds, hexahydropyrrolo[1,2-*a*]pyrazine-1,4-dione and 2,3-indolinedione, might be the intermediate metabolites of some of the predicted secondary metabolite gene clusters. However, the biosynthetic pathways of the two compounds in *Shewanella* sp. Lzh-2 have not been characterized.

### Data availability.

This whole-genome shotgun project has been deposited in GenBank under the accession no. PRJNA694369. The raw data of genome sequencing have been deposited in the Sequence Read Archive (SRA) database under the accession no. SRP309231. The version described in this paper is the first version.
